# Umgang mit Bissverletzungen

**DOI:** 10.1007/s00104-024-02173-z

**Published:** 2024-09-24

**Authors:** Daniel Vergote, Martin Mentzel, Myriam Andreas, Richard-Tobias Moeller, Simon Bauknecht

**Affiliations:** Klinik für Unfall‑, Hand‑, Plastische und Wiederherstellungschirurgie, Universitätsklinikum Ulm, Albert-Einstein-Allee 23, 89081 Ulm, Deutschland

**Keywords:** Tierbisse, Kontaminierte Wunde, Infektion, Keimspektrum, Hand, Animal bite, Contaminated wounds, Infection, Spectrum of germs, Hand

## Abstract

Bissverletzungen verursachen kontaminierte Wunden mit großem Infektionsrisiko. Es handelt sich um ein progredientes Geschehen. Die Prognose hängt weniger von der Ausdehnung an der Körperoberfläche als vielmehr von der Eindringtiefe ab. Aufgrund enger räumlicher Lagebeziehungen sind die Strukturen an der Hand bei Bissverletzungen besonders gefährdet. Wir werten in dieser Arbeit die Dynamik des Krankheitsverlaufs in Abhängigkeit des Verletzungsausmaßes und des Therapiebeginns aus. Insgesamt 332 Bissverletzungen wurden behandelt und retrospektiv ausgewertet. Die Einteilung der Verletzungsschwere richtete sich nach den betroffenen Kompartimenten in fünf Grade. Die Auswertung der Therapie erfolgte gemäß ihrem zeitlichen Verlauf in vier Gruppen. Die Infektraten stiegen mit dem Schweregrad der Verletzung und der verstreichenden Zeit. Bestanden am Unfalltag bei 11 % der Patienten Infektzeichen, waren es am Folgetag unter konservativer Anbehandlung bereits 72 % bzw. 93 %, wenn bis dahin keine Therapie erfolgte. Auf diesem Niveau bewegten sich auch die auswärts konservativ anbehandelten Patienten. Auch die durchschnittliche Zahl der Eingriffe stieg mit dem Intervall zwischen Biss und Versorgungsbeginn. Biss ist nicht Biss. Auf das Ausmaß kommt es an. Kutane Verletzungen entwickeln in 23 % der Fälle Infektzeichen, können aber meist konservativ behandelt werden. Alle tieferen Verletzungen bedürfen einer chirurgischen Therapie mit Débridement, Ruhigstellung und Antibiose. Bei unmittelbarer chirurgischer Therapie waren Revisionen in 4 % notwendig.

## Abstract (en)

Bite injuries cause contaminated wounds with a high risk of infection. It is a progressive process. The prognosis depends less on the extent of the surface area but more on the depth of penetration. Due to the close spatial relationships the structures in the hand are particularly at risk from bite injuries. This study evaluated the dynamics of the disease progression based on the extent of injury and the timing of treatment initiation. A total of 332 bite injuries were treated and retrospectively analyzed. The severity of injuries was classified into five grades based on the affected compartments. The evaluation of the treatment was conducted according to the timing in four groups. The infection rates increased with the severity of the injury and the elapsed time. On the day of the accident 11% of patients showed signs of infection, rising to 72% the following day under conservative treatment, and 93% if no treatment was initiated by then. Patients treated conservatively elsewhere also showed similar levels. The average number of interventions also increased with the interval between the bite and the start of treatment. Not all bites are equal. The extent matters. Cutaneous injuries show signs of infection in 23% of cases but can mostly be treated conservatively. All deeper injuries require surgical treatment with debridement, immobilization and antibiotics. After immediate surgical treatment revision was necessary in 4% of cases.

## Hintergrund

In deutschen Haushalten lebten 2022 ca. 34,4 Mio. Hunde, Katzen, Kleinsäuger und Ziervögel. Katzen nehmen mit 15,2 Mio. hierbei den größten Anteil ein [1]. Bissverletzungen, meist durch das eigene Haustier [2], treten mit 30.000 bis 50.000 Fällen in Deutschland häufig auf und da viele Patienten zunächst keinen Arzt konsultieren, ist von einer hohen Dunkelziffer auszugehen. Schätzungen zufolge verlaufen 80 % aller Tierbissverletzungen unkompliziert und werden nicht medizinisch versorgt [3].

Medizinisch dokumentiert werden am häufigsten Hundebisse, gefolgt von Katzenbissverletzungen. Die meisten Bisse finden sich an der oberen Extremität, insbesondere distal an der Hand und der Finger [4]. An der Hand befinden sich die einzelnen Organstrukturen in enger räumlicher Beziehung und sind sehr zierlich. Aufgrund des Verletzungsmusters bei Bissen und der vielseitigen bakteriellen Flora in tierischer und menschlicher Mundflora können bereits kleinste Bissverletzungen an der Hand zu schwerwiegenden Verläufen mit fulminanten Infektionen führen. Während bei Hundebissen häufig offene, klaffende Wunden mit ausgeprägtem Weichgewebsschaden entstehen, führen Katzenbisse zu tiefreichenden Wunden, die von außen oftmals harmlos imponieren. Hundebisse gehen aufgrund der enormen Krafteinwirkung mit höheren Verletzungsfolgen für funktionelle Strukturen einher, eine ärztliche Vorstellung ist dann unabdingbar. Bei Katzenbissen hingegen kommt es in der Regel nur zu milde ausgeprägten Weichgewebsschäden. Patienten suchen daher nicht unmittelbar eine chirurgische Notaufnahme auf. Durch die nadelscharfen Zähne gelangen jedoch zahlreiche Bakterien zu tiefreichenden Strukturen. Die meist rasch verschlossene Bisswunde lässt einen serösen Ablauf nicht zu, sodass es in der Tiefe zum schnellen Wachstum vor allem anaerober und fakultativ anaerober Bakterien kommt [5]. Dies führt zu einem bis zu 6‑fach höheren Infektionsrisiko im Vergleich zu Hundebissen [6]. Katzenbissverletzungen wird daher ein hohes Infektionsrisiko von 10–50 % zugerechnet [7, 8].

**Abb. 1:**
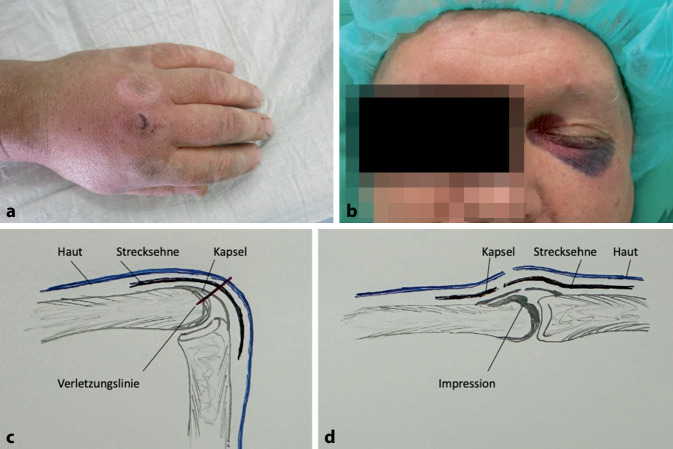
**a** Klinisches Bild nach „Kampfbiss“. **b** Typische Begleitverletzung mit Monokelhämatom bei handfester Auseinandersetzung. **c** Moment des Faustschlags. **d** Untersuchungsbefund mit Kulissenphänomen in Streckstellung

Menschenbisse treten überwiegend im Rahmen körperlicher Auseinandersetzungen auf. Sogenannte Kampfbisse entstehen durch den Schlag einer geballten Faust auf die Zähne einer anderen Person. Das Grundgelenk wird hierbei am häufigsten verletzt. Die darüber liegende Haut ist bei geballter Haut straff gespannt und bietet wenig Weichgewebsschutz. Oftmals kommt es zur Verletzung des Streckapparats und Gelenkeröffnung [9]. Bei der Versorgung derartiger Verletzungen muss der „Kulisseneffekt“ beachtet werden (Abb. 1). Bei entspannter Handstellung kommt es durch Retraktion der Strecksehne zum Verschwinden der tieferen Verletzung aus dem Sichtfeld und durch das Zulappen der Gelenkkapsel kann sich durch schnelles Keimwachstum eine septische Arthritis entwickeln [10].

Auch exotische Bissverletzungen verzeichnen in Europa einen Inzidenzanstieg. Weltweit treten jährlich über 5 Mio. Vergiftungen durch Schlangenbisse auf, davon verlaufen ca. 125.000 tödlich [11]. Diese kommen vor allem in Afrika, Südostasien und Lateinamerika vor. Da Reptilien auch in Europa vermehrt als Haustier gehalten werden, finden sich auch in europäischen Notaufnahmen vermehrt Bissfolgen [12]. Durch die injizierten Toxine kommt es aufgrund der antimikrobiellen Wirkung seltener zu Infektionen, jedoch können schwerwiegende Verläufe durch lokale und systemische Inflammationsreaktionen entstehen [13].

Die Daten in der Literatur legen nahe, dass eine angemessene Wundversorgung der wichtigste Faktor in der Prävention von Infektionen nach Bissverletzungen darstellt [14–16]. Das vollständige Ausmaß der Verletzung ist allein durch die chirurgische Exploration möglich. Bestehen bereits Zeichen einer Infektion, besteht in der Regel die Indikation zur chirurgischen Intervention. In dieser Studie stellen wir Behandlungsverläufe nach Bissverletzungen dar und arbeiten insbesondere die Entwicklung von Infektionen und die Notwendigkeit von Folgeeingriffen in Abhängigkeit der zeitlichen Versorgung auf.

## Material und Methoden

Im Zeitraum von 2009 bis 2021 wurden in der Sektion für Hand‑, Plastische und Wiederherstellungschirurgie des Universitätsklinikums Ulm 332 Patienten mit Bissverletzungen behandelt. Die aus der Krankenakte gewonnenen Daten umfassen patientenspezifische Daten, die Verletzung verursachende Spezies, Lokalisation der Bissverletzung, Zeitpunkt der ärztlichen Vorstellung und Verlauf der Therapie.

Die Einteilung verschiedener Variablen wurde wie folgt durchgeführt:

Die medizinische Versorgung wurde unterschieden je nach ärztlicher Erstvorstellung und Vorbehandlung. Die Versorgung erfolgte entsprechend:

- Primär: Am Tag der Bissverletzung stellte sich der Patient in unserer Notaufnahme vor.
- Verzögert primär: Die Vorstellung erfolgte als Erstvorstellung in unserer Notaufnahme, jedoch verzögert ab dem 1. posttraumatischen Tag.
- Sekundär: Die Vorstellung erfolgte zunächst beim Hausarzt oder in einer externen Klinik, eine konservative Therapie wurde eingeleitet. Im weiteren Verlauf erfolgte die Überweisung in unsere Abteilung.
- Sekundärversorgung: Ein chirurgisches Débridement erfolgte in einer externen Klinik, im Verlauf wurde der Patient in unserer Abteilung vorstellig.

Beim Vorliegen der klassischen Entzündungszeichen Rubor, Calor, Dolor, Tumor und Functio laesa wurde der Fall als „Infektion“ gewertet. Die Verletzung verursachende Spezies wurden in sechs Gruppen eingeteilt. Es ergaben sich Bissverletzungen durch Katzen, Hunde, Wildtiere (Affe, Schlage, Fisch, Fuchs, Schaf, Wildschwein), Nagetiere (Meerschweinchen, Hamster, Ratte, Biber), Großtiere (Pferd, Esel) und Menschen. Die Lokalisationsangabe der Bissverletzung teilte sich ein in Finger (Grundgelenk bis Fingerspitze), Mittelhand mit Handwurzel und Handgelenk, Arm, Bein und Gesicht.

Die Eindringtiefe der Bissverletzung wurde anhand der Beschreibung des klinischen Befunds und des Operationsberichts dokumentiert. Dabei erfolgte die Klassifizierung in fünf Schweregrade:

- Grad I: oberflächliche Hautverletzung, die Epi- oder Dermis betreffend,
- Grad II: Eindringtiefe bis in die Subkutis,
- Grad III: tiefe Verletzung mit Verletzung der Faszie oder Sehnenscheide,
- Grad IV: Verletzung funktioneller Strukturen (Sehnen, Muskeln, Gefäße oder Nerven),
- Grad V: Verletzung von Knochen oder Eröffnung von Gelenken.

Lagen mehrere Bissverletzungen vor, wurde in unsere Auswertung stets der Biss mit der tiefsten Eindringtiefe in die Auswertung aufgenommen.

Die Dokumentation der Daten und Auswertung erfolgte mithilfe von Microsoft Excel (Version 16.66.1) und IBM SPSS Statistics (Version 28.01.1). Deskriptive Statistik wurde verwendet, um Häufigkeiten darzulegen und Merkmale zu beschreiben. Kreuztabellen wurden erstellt, um Beziehungen zwischen unterschiedlichen Variablen zu untersuchen und darzustellen.

Zur Diskussion der Ergebnisse erfolgte eine Literaturrecherche in den elektronischen Datenbanken von PubMed, Google Scholar und Medline.

## Ergebnisse

Einen Vergleich der Bissverletzungen nach verursachender Spezies hinsichtlich acht Kategorien zeigt Tab. 1. Dabei waren 214 Patienten weiblich (64 %) und 118 Patienten männlich (36 %). Das mediane Alter betrug 44,5 Jahre (Min. 1, Max. 94). Zunächst fällt auf, dass 91 % der Verletzungen durch Katzen (55 %) und Hunde (36 %) verursacht wurden. Wildtiere, Nagetiere, Großtiere und der Mensch teilten sich die übrigen 9 %. Katzenbisse wiesen im Mittel mit 77 % die höchste Infektrate auf. Während von Katzen- und Hundebissen vorwiegend Menschen in mittlerem Lebensalter betroffen waren, überwogen bei Nage- und Großtieren Kinder und Jugendlich. Durch Wildtiere und Menschen waren erwartungsgemäß zumeist junge Erwachsene gefährdet. Katzen‑, Hunde- und Menschenbisse erreichten meist die Mittelhand, die übrigen Spezies fast nur die Finger. Die Mehrzahl der Katzen‑, Hunde- und Nagetierbisse betrafen die Subkutis, Großtier- und Menschenbisse meist Knochen und Gelenke. Bei den Wildtieren war die Bilanz ausgeglichen.

**Tab. 1:** Kollektivgesamtübersicht

Kategorie		Bissverursachende Spezies					
		Katze, Hund (*n* = 184, 55,4 %) (*n* = 118, 35,5 %) (%)		Wildtier (*n* = 8, 2,4 %) (%)	Nagetier (*n* = 13, 3,9 %) (%)	Großtier (*n* = 3, 0,9 %) (%)	Mensch (*n* = 6, 1,8 %) (%)
Infektion	*Ja* (*n* = 227, 68,4 %)	77	56	75	62	67	67
	*Nein* (*n* = 105, 31,6 %)	23	44	25	38	33	33
Alter	*Bis 20 Jahre* (*n* = 51, 15,4 %)	13	14	25	46	67	33
	*21–40 Jahre* (*n* = 92, 27,7 %)	27	26	50	31	–	50
	*41–60 Jahre* (*n* = 134, 40,4 %)	42	45	–	23	–	17
	*61–80 Jahre* (*n* = 47, 14,1 % )	16	13	25	–	33	–
	*Über 80 Jahre* (*n* = 8, 2,4 %)	3	3	–	–	–	–
Lokalisation	*Finger* (*n* = 124, 37,3 %)	34	34	100	69	67	33
	*Mittelhand* (*n* = 175, 52,7 %)	57	53	–	23	33	67
	*Arm* (*n* = 27, 8,1 %)	9	8	–	8	–	–
	*Bein* (*n* = 2, 0,6 %)	–	2	–	–	–	–
	*Gesicht* (*n* = 4, 1,2 %)	–	3	–	–	–	–
Wundtiefes	*Grad I* (*n* = 34, 10,2 %)	14	6	–	15	–	–
	*Grad II* (*n* = 127, 38,2 %)	42	36	38	38	–	–
	*Grad III* (*n* = 59, 17,8 %)	20	17	13	8	33	–
	*Grad IV* (*n* = 65, 19,6 %)	19	19	13	23	–	50
	*Grad V* (*n* = 47, 14,2 %)	6	22	38	15	67	50
Keimnachweis	*Ja* (*n* = 139, 41,9 %)	52	26	13	46	67	50
	*Nein* (*n* = 135, 40,7 %)	34	51	50	38	33	33
	*Kein Abstrich* (*n* = 58, 17,4 %)	14	23	38	15	–	17
Versorgung	*Primär* (*n* = 112, 33,7 %)	26	46	50	31	33	33
	*Verzögert primär* (*n* = 120, 36,2 %)	48	22	–	23	33	33
	*Sekundär* (*n* = 81, 24,4 %)	23	25	38	31	33	–
	*Sekundärversorgung* (*n* = 19, 5,7 %)	3	7	13	15	–	33
Behandlung	*Ambulant* (*n* = 43, 13 %)	13	13	13	15	–	33
	*Stationär* (*n* = 289, 87 %)	87	87	88	85	100	67
Stationär	*0 Tage* (*n* = 43, 13 %)	13	13	13	15	–	33
	*1–5 Tage* (*n* = 191, 57,5 %)	61	55	63	62	–	17
	*6–10 Tage* (*n* = 69, 20,8 %)	18	24	13	23	67	33
	*11–15 Tage* (*n* = 15, 4,5 %)	5	5	–	–	–	–
	*>* *15 Tage* (*n* = 13, 3,9 %)	3	3	13	–	33	17

Katzenbisspatienten stellten sich meist verzögert primär vor, während sich Patienten mit Hunde- und Wildtierbissen eher primär vorstellten. Auffällig war weiterhin die hohe Rate auswärts voroperierter Menschenbisse, die dann im Verlauf wegen Heilungsstörungen zu uns überwiesen wurden.

Die Infektraten stiegen mit dem Schweregrad der Verletzung und beliefen sich auf folgende Prozentsätze: Grad I: 24 %; Grad II: 72 %; Grad III: 81 %; Grad IV: 80 %; Grad V: 57 %. I°-Verletzungen wurden dabei stets konservativ behandelt.

**Abb. 2:**
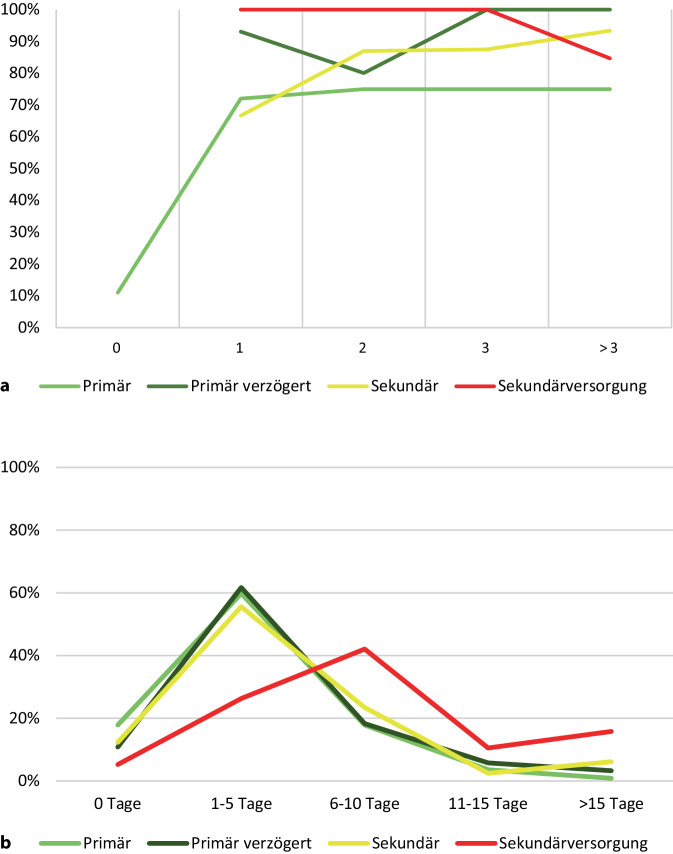
**a** Infektionsrate in Prozent je nach Zeitpunkt der operativen Versorgung in Tagen (0 entspricht dabei dem Tag der Verletzung) und Art der Versorgung. **b** Dauer der stationären Behandlung je nach Versorgung (0 Tage entspricht dabei einer ambulanten Therapie)

Insgesamt 112 Patienten stellten sich am Tag der Bissverletzung in unserer Notaufnahme vor. Am Unfalltag bestanden bei 11 % der Patienten Infektzeichen, am Folgetag unter konservativer Therapie bereits 72 %. Einen ähnlichen Wert präsentierte das Kollektiv der Patienten, die extern eine Initiierung der konservativen Therapie erhielten. 67 % wiesen am Tag nach der Bissverletzung Infektzeichen auf. Mit Verzögerung der operativen Therapie stieg die Rate der Infekte stetig an. Patienten, die sich verzögert ärztlich vorstellten, zeigten am 1. Tag nach der Bissverletzung bereits in 93 % der Fälle Infektzeichen, ab dem 3. Tag in 100 % (Abb. 2a). Im Durchschnitt wurden 1,1 Operationen notwendig bei primärer Versorgung nach im Schnitt einem Tag und 1,3 Operationen bei sekundärer Versorgung nach im Schnitt 5 Tagen.

Insgesamt 19 Patienten stellten sich durchschnittlich nach 2 Tagen zunächst in einer externen Klinik vor, wurden dort chirurgisch versorgt und im Verlauf in unsere Klinik überwiesen. Die Revision in unserer Klinik erfolgte dann im Schnitt am 16. Tag nach der Bissverletzung. Bei 17 Patienten (90 %) erfolgte die Revision bei einem persistierenden Infekt, bei 2 Patienten zur weiteren Therapie mittels plastischer Deckung. Im Schnitt wurden 2,1 operative Eingriffe notwendig.

Ein Keimnachweis gelang bei 42 % aller Patienten, in etwas mehr als der Hälfte lag dann eine Mischinfektion mit bis zu fünf Erregern vor. Drei Viertel der mikrobiologischen Nachweise waren Bakterien der Gattung *Pasteurella* gefolgt von Fusobakterien und Streptokokken. Nahezu alle Patienten (98 %) wurden antibiotisch therapiert. In über 80 % wurde eine kalkulierte Antibiose mit Cefuroxim 1,5 g intravenös begonnen und in knapp 7 % der Patientenfälle wurde die Antibiotikatherapie im Verlauf antibiogrammgerecht umgestellt.

Insgesamt 22 % der Patienten wiesen keine Vorerkrankungen auf, 26 % der Patienten waren Raucher, bei 16 % der Patienten bestand eine arterielle Hypertonie, bei 6 % ein Diabetes mellitus und bei 5 % eine Hypothyreose.

Die Länge der stationären Behandlung unterscheidet sich wesentlich nur im Kollektiv „Sekundärversorgung“. Am häufigsten war die stationäre Behandlung zwischen 6 und 10 Tagen notwendig und bei knapp einem Viertel der Patienten sogar über 15 Tagen. In den anderen Kollektiven war die stationäre Behandlung jeweils in knapp 60 % der Fälle für 1 bis 5 Tage notwendig. Eine ambulante Therapie wurde am häufigsten im Kollektiv der „Primärversorgung“ verzeichnet (Abb. 2b).

## Diskussion

Bei Betrachtung des Kollektivs fällt der hohe Anteil an Bissverletzungen durch Katzen und die überwiegende Lokalisation an der Hand (90 %) auf. Während allgemein Hunde als häufigste Ursache für Bissverletzungen gelten [17, 18] und eine Verletzung der Hand in 39–75 % der Fälle vorliegt [19], erfolgte in unserem Kollektiv eine Vorselektion aufgrund der Versorgung durch die Sektion der Handchirurgie.

Die retrospektive Auswertung von 332 Bissverletzungen zeigt die steigenden Infektraten in Abhängigkeit vom Ausmaß der Verletzung und der Zeit, die bis zum Therapiebeginn verstreicht. Biss ist nicht Biss und Infekt ist nicht Infekt. Auf das Ausmaß kommt es an. Rein kutane Verletzungen entwickelten in 23 % Infektzeichen. Eine konservative Therapie war ausreichend. Alle tieferen Verletzungen führten im Verlauf der ersten Tage in über 80 % zu Wundinfektionen. Sie bedurften einer chirurgischen Therapie mit umfassendem Débridement, ggf. Drainageneinlage, Ruhigstellung und flankierender Antibiose. Unter diesem Vorgehen ließen sich Bissverletzungen bis zum 5. Tag in der Regel mit einer Operation sanieren. Bei unmittelbarer chirurgischer Versorgung waren nur in 4 % Revisionen erforderlich. Dabei ist zu berücksichtigen, dass ausgeprägte Befunde an tiefen Strukturen wie Sehnen und Gelenke häufig einer geplanten Revision im Intervall bedürfen.

Zieht man die verschiedenen Schweregrade von Bissverletzungen in Betracht und berücksichtigt die zeitliche Dynamik eines Infektionsgeschehens, wird klar, dass pauschale Angaben über Infektraten, wie sie in der Literatur zwischen 15–68 % [17, 20, 21] angegeben werden, für die Entscheidung im Einzelfall wenig hilfreich sind.

Die Beurteilung des Ausmaßes einer Bissverletzung ist abgesehen von ganz oberflächlichen Kratzern und Läsionen nicht immer einfach. Die Anamnese (Spezies, Unfallhergang) gibt Anhaltspunkte. Die klinische Untersuchung (klassische Infektzeichen, Druck‑, Dehnungs- und Bewegungsschmerzen) gibt wie das Röntgenbild (Fraktur, Fremdkörper) Aufschlüsse. Nichts geht jedoch über die chirurgische Exploration, die sowieso für jede Verletzung obligat ist.

**Abb. 3:**
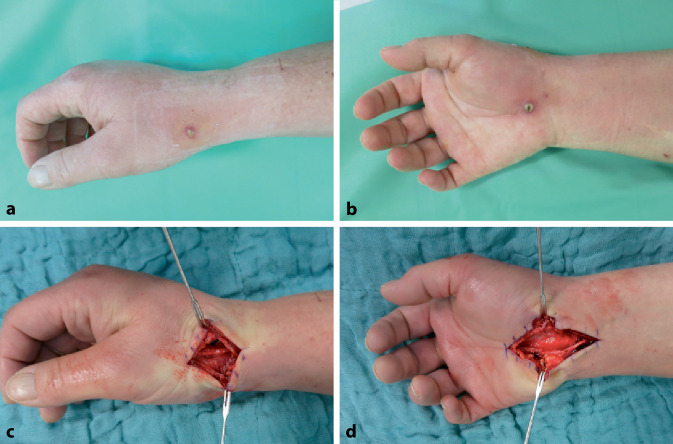
Klinisches Bild bei Katzenbissverletzung: **a**, **b** Bissverletzung radial und palmar auf Höhe der Handwurzel mit wenige Millimeter messender Wunde. **c**, **d** Chirurgische Exploration mit Eröffnung des Karpaldachs

Hierzu sollte die Hautinzision angemessen verlängert und die Bisswunde ausgeschnitten werden. Anschließend erfolgt ein ausführliches Débridement und Nekrosektomie zur Reduktion der Keimzahl und Optimierung der Durchblutung des Weichgewebes (Abb. 3).

Komplizierte Verlaufsformen mit schweren Infektionen gehen, wie auch in der Literatur beschrieben, nahezu immer von den subkutan gelegenen Faszienstrukturen aus. Die chirurgische Exploration muss eine Übersicht über alle verletzten Strukturen ermöglichen [22]. Der primäre Wundverschluss von Bissverletzungen im Gesicht wird einheitlich in der Literatur beschrieben und geht mit guten ästhetischen und funktionellen Ergebnissen einher [23–25]. Der primäre Verschluss an anderen Lokalisationen wird in der Literatur kontrovers diskutiert [3, 4, 26]. In unserem Fall erfolgte in 84 % ein Wundverschluss über eine Drainage mit situativen Hautnähten.

Die chirurgische Exploration ist am Unfalltag, an dem wir es in der Regel nur mit kontaminierten Wunden ohne Infektzeichen zu tun haben, einfacher als an den Folgetagen, wenn bereits Infekte vorliegen.

Der Faktor Zeit spielt eine wichtige Rolle bei der Behandlung von Bissverletzungen. Die Infektraten von Katzenbissen liegen höher als die der Hundebisse, obwohl ihr Weichgewebstrauma geringer ist. Genau deswegen werden sie unterschätzt, und die Patienten stellen sich erst verspätet vor. Patienten mit Hundebissen nehmen aufgrund des großflächigeren Weichgewebstraumas (vielleicht auch aus juristischen Überlegungen) in der Regel bereits am Unfalltag ärztliche Hilfe in Anspruch und je größer die Wundfläche, desto schneller wird chirurgisch interveniert.

Während in der Literatur ein erhöhtes Risiko für Patienten mit Herz-Kreislauf-Erkrankungen/arterieller Hypertonie, Diabetes oder Lungenerkrankungen dokumentiert wurde, findet sich in unserem Kollektiv diesbezüglich kein nachweisbarer Zusammenhang [17].

Eine Antibiose erfolgt flankierend. In unserem Kollektiv kam in 80 % der Fälle entsprechend der damaligen Empfehlung Cefuroxim zum Einsatz. Beim Vergleich mit der Arbeit von Kuhbier et al. – hier erhielten über 75 % der Patienten eine Therapie mit Sultamicillin (Prodrug, die im Körper zu Ampicillin und Sulbactam umgewandelt wird; [17]) – fällt jedoch kein erhöhtes Infektionsrisiko auf. Dies unterstreicht den Stellenwert der chirurgischen Therapie.

Bakterien der Gattung *Pasteurella* waren die am häufigsten isolierten Mikroorganismen. Auch in der Literatur gilt insbesondere das fakultativ anaerobe Stäbchenbakterium *Pasteurella multocida* als das am häufigsten nachgewiesene Bakterium nach Katzen- und Hundebissen und findet sich in mehr als 50 % aller infizierten Wunden [19, 27]. Typischerweise ist das Erregerspektrum in Bisswunden polymikrobiell.

Während bei jeder Bissverletzung mit Zeichen einer Infektion die kalkulierte Antibiotikatherapie als obligat gilt, ist die aktuelle Studienlage zur prophylaktischen Antibiotikatherapie nicht einheitlich und gestattet keine eindeutige Aussage. Eine Cochrane-Analyse empfiehlt die prophylaktische Behandlung für Bisse an der Hand und für Menschenbisse [28]. Da das Ausmaß und die Tiefe der Bissverletzung nicht immer richtig eingeschätzt werden, ist die Indikation zur kurzzeitigen Antibiotikaprophylaxe für 3 bis 5 Tage großzügig zu stellen. Die aktuellen Empfehlungen zur Antibiotikatherapie werden in Tab. 2 dargestellt.

**Tab. 2:** Antibiotische Therapie bei Bissverletzungen

	1. Wahl	Dosierung	Alternative	Dosierung
*Hunde- und Katzenbiss*				
Prophylaxe/Kalkuliert	Amoxicillin/Clavulansäure Ampicillin/Sulbactam	2 × 875/125 mg p.o. 3 × 3 g i.v.	Cefuroxim + Clindamycin/Metronidazol	3 × 1,5 g i.v. + 3 × 0,6 g/3 × 0,5 g i.v.
V. a. MRSA	Linezolid	2 × 0,6 g i.v.	–	–
*Menschenbiss*	Amoxicillin/Clavulansäure Ampicillin/Sulbactam	2 × 875/125 mg p.o. 3 × 3 g i.v.	Ertapenem	1 × 1 g i.v.
*Resistenzgerecht*	Nach Antibiogramm	Nach Sensibilität	–	–

Grundsätzlich ist jedoch auch eine Übertragung anderer Infektionserreger wie beispielsweise HBV/HCV, HIV oder *Candida* spp. nicht zu vergessen. Es sollte der Impfstatus entsprechend den Empfehlungen der ständigen Impfkommission des Robert Koch-Instituts abgefragt werden. Bei sauberen, geringfügigen Wunden ist die Tetanusimmunprophylaxe notwendig, wenn die vollständige Tetanusimmunisierung länger als 10 Jahre zurückliegt. Bei größeren oder verschmutzen Wunden sollte dieser Zeitraum nicht länger als 5 Jahre betragen. Des Weiteren ist das Risiko einer Tollwutexposition zu prüfen. Besteht der Verdacht, sollte das Gesundheitsamt hinzugezogen werden, denn es besteht Meldepflicht. Die ausgiebige Wundreinigung und antiseptische Spülung ist essenziell und eine postexpositionelle Immunprophylaxe muss erwogen werden. Die Schutzwirkung einer unverzüglich nach Exposition und lege artis durchgeführten Postexpositionsprophylaxe liegt bei Immungesunden bei nahezu 100 % [29].

## Fazit für die Praxis

- Jede Bissverletzung kann zu einer schweren Infektion führen.
- Die chirurgische Therapie mit Débridement und Spülung stellt den Hauptpfeiler in der Behandlung dar.
- Eine frühzeitige Therapie kann dabei eine Infektionsentwicklung und notwendige Revisionen vermeiden.

## Data Availability

Die erhobenen Datensätze können auf begründete Anfrage in anonymisierter Form beim korrespondierenden Autor angefordert werden.
